# Conventional and Emerging Surgical and Non-surgical Approaches to the Management of Skin Burn Scars: A Comprehensive Review

**DOI:** 10.7759/cureus.90982

**Published:** 2025-08-25

**Authors:** Yozahandy A Abarca-Pineda, Ian C Alaniz, Amr Ibraheam, Ira Bhasin, Saya Alasaadi, Miguel Linares García, Janani Suresh, Long Yin Cai, Humza F Siddiqui

**Affiliations:** 1 Department of Internal Medicine, Hospital Médica Sur, Mexico City, MEX; 2 Department of Internal Medicine, TecSalud School of Medicine and Health Sciences, Tecnológico de Monterrey, Mexico City, MEX; 3 Department of General Practice, Universidad Panamericana, Mexico City, MEX; 4 Department of Internal Medicine, St. George's University School of Medicine, St. George's, GRD; 5 Department of Internal Medicine, Kasturba Medical College, Mangalore, IND; 6 Department of Medicine, University College Dublin, Dublin, IRL; 7 Department of Internal Medicine, Hospital Materno Infantil José Domingo De Obaldía, Chiriquí, PAN; 8 Department of Dermatology, Queen's Hospital, Barking, Havering and Redbridge University Hospitals NHS Trust, London, GBR; 9 Department of Internal Medicine, Caribbean Medical University, Willemstad, CUW; 10 Department of Internal Medicine, Jinnah Postgraduate Medical Centre, Karachi, PAK

**Keywords:** burn scars, full thickness skin graft, hypertrophic scars, keloids, split-thickness skin graft, topical corticosteroids (tcs)

## Abstract

Burn cases pose a significant burden on the healthcare system worldwide. The acute loss of the barrier function of the skin can lead to severe shock and sepsis. Burn wounds can evolve into hypertrophic scars and keloids that impair function and aesthetic appearance. Early intervention with both non-surgical and surgical management strategies is crucial to curtail severe consequences. This review aims to provide a comprehensive assessment of treatment modalities ranging from topical and intralesional therapies to surgical procedures, while also highlighting the role of adjunct and emerging strategies. Topical emollients, corticosteroid creams and injections, and silicone gel help restore barrier function and reduce inflammation in patients with superficial or partial-thickness burn injuries. Autologous and allogenic grafts and flaps have proven effective in restoring function and increasing patient satisfaction in treating deep partial-thickness and full-thickness burns. However, the appropriate timing for employing these conventional therapies to gain maximum benefit remains debatable and can result in undesirable complications. Adjunct therapies, including microneedling, platelet-rich plasma (PRP), laser, and physical therapy, have shown mixed results in improving outcomes. Newly emerging techniques, such as stem cells, nanomedicine, and 3D bioprinting, have demonstrated promising potential in enhancing patient outcomes. Nonetheless, large clinical trials with human participants are needed before these modalities can be widely implemented in clinical practice without reservations. It is imperative to establish definitive treatment guidelines and explore advanced avenues to further augment the quality of life among burn victims.

## Introduction and background

Burn injuries remain a significant global health burden, affecting millions of patients annually. They lead to several long-term complications that impact activities of daily living, occupational function, and psychological well-being [1]. Despite being largely preventable, approximately 11 million people worldwide require medical treatment for burns each year, according to the WHO. Over 180,000 burn-related deaths occur annually in low- and middle-income countries [2]. Most burn cases occur in home or workplace settings, with adult women and children being the most common victims. The most common causes of burns include exposure to flames, hot liquids, caustic chemicals, electricity, and radiation [3]. Burns cause loss of the skin’s barrier function, increasing fluid loss and pathogen invasion, which can lead to severe consequences, including sepsis and shock [1, 2]. Although advances in acute burn care have significantly improved survival rates, post-burn scarring remains a major challenge. Up to 70% of burn patients develop hypertrophic scars or contractures, resulting in chronic pain, reduced mobility, and functional limitations [4]. The psychosocial consequences of burn scars are profound, often resulting in anxiety, depression, post-traumatic stress disorder (PTSD), and social stigmatization [5]. Burn patients are prone to visible scarring, which significantly impacts self-esteem, body image perception, and quality of life, particularly among pediatric and adolescent populations [6]. Additionally, burn survivors frequently encounter occupational challenges due to physical impairments, prolonged recovery, and societal biases, further exacerbating their financial and emotional distress [7].

Effective scar management remains complex and multifaceted despite advancements in burn care. Current treatment modalities are broadly categorized into non-surgical and surgical interventions. Early wound care and infection control play a critical role in minimizing excessive scarring [8]. Non-surgical management includes topical and intralesional therapies such as silicone gel, corticosteroids, botulinum toxin, and emollients [9]. Recently, laser therapy, microneedling, platelet-rich plasma (PRP), and cryotherapy have emerged as adjunctive treatment options [10]. For more severe burn scars causing functional impairment, surgical interventions such as scar excision, Z-plasty, skin grafting, and flap reconstruction remain essential [9, 11]. Recent innovations in burn scar management focus on regenerative medicine, including bioengineered skin substitutes, stem cell therapy, and nanomedicine applications, offering promising alternatives to traditional treatments. Personalized treatment approaches and long-term follow-up are essential to ensure optimal functional and aesthetic outcomes for patients [2, 12].

This review aims to provide a comprehensive analysis of burn scar assessment methodologies, classification, and current management strategies. It incorporates both well-established and emerging therapeutic approaches while discussing the latest advancements in treatment. It is imperative to underscore the importance of a multidisciplinary approach to improve the long-term quality of life of burn survivors and minimize recovery time.

## Review

Classification of burn wound

**Figure 1:**
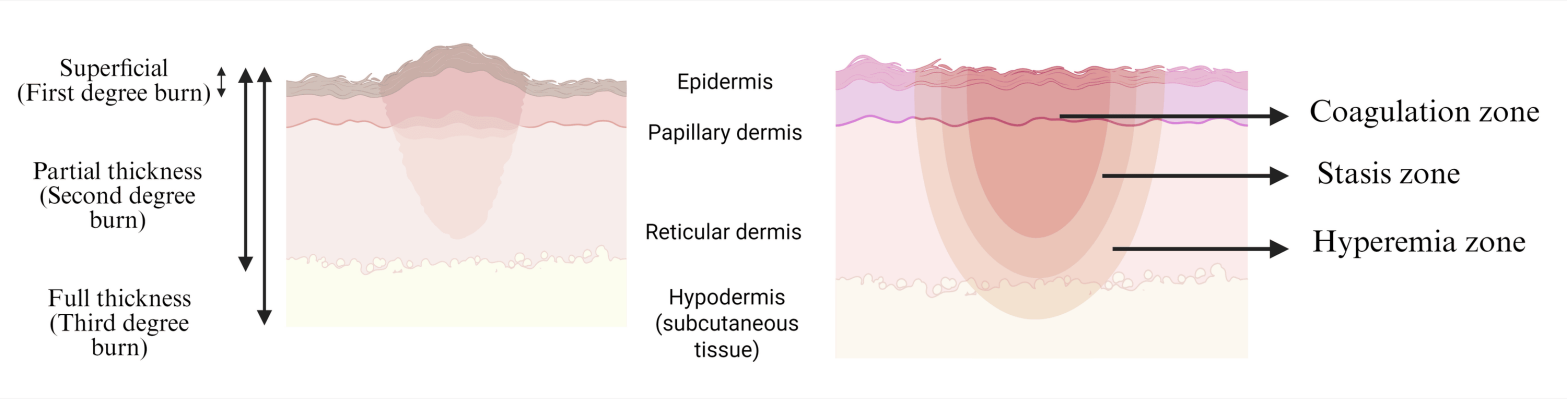
Classification of burn injury. Image Credit: Figure created by Humza Siddiqui using BioRender.com.

Scars are formed as a result of the dynamic and complex phenomenon of tissue repair. Various stimuli alter the normal process of tissue repair and cause abnormal tissue healing, resulting in scar formation. Factors affecting burn lesion repair include hypoxia, edema, the amount of necrotic tissue, and an intense inflammatory reaction [13]. It is important to assess the severity of these scars to establish a plan of action and effectively treat these lesions. This is indicated by the depth and size of the burn. Burn size is assessed by measuring the total body surface area (TBSA) of the lesion. Burn depth is measured by assessing the dermal layers affected by the burn injury. Burn scars are widely classified into three main categories: superficial partial-thickness, deep partial-thickness, and full-thickness burns. This classification is based on the increasing depth of the scar tissue [14, 15]. The burn scar is also divided into three zones: the coagulation zone, the stasis zone, and the hyperemia zone. The coagulation zone is the central region of the burn that is irreversibly damaged due to protein coagulation. The stasis zone surrounds the central necrotic region and is characterized by reduced blood flow, potentially leading to ischemia. The stasis zone is a key target during management, as it is salvageable and its viability impacts the overall outcome. The hyperemia zone is the outermost layer, which exhibits increased blood flow due to vasodilation and generally recovers without severe consequences [1, 4, 14]. Table 1 provides an overview of burn injury thickness and Figure 1 describes the classification of burn injury.

**Table 1:** Overview of burn thickness. Table Credits: Table created by the authors using references [13-15].

Burn injury thickness	Layers involved	Comment on healing
Superficial partial-thickness burns	Epidermal layer and the superficial part of the dermis	Can heal spontaneously
Deep partial-thickness burns	Epidermis and the majority of the dermis, with damage to deeper skin structures (blood vessels, nerves, and hair follicles)	May heal spontaneously, but often requires medical intervention depending on severity
Full-thickness burns	All layers of the skin are involved; there may also be damage to subdermal structures (muscle, cartilage, or bone)	Cannot heal spontaneously due to absence of viable epidermal appendages at the base of the wound

Evaluation of acute burn injury

The Patient and Observer Scar Assessment Scale (POSAS), Lund-Browder Assessment Chart, Lund-Browder Modified Chart, and VSS are noteworthy tools used to assess burn injuries. The Lund-Browder chart is widely used and relatively easy to apply for measuring the burned TBSA. The Lund-Browder Chart is a pictorial representation used to determine the burn area and is considered the most accurate method of identifying the affected surface area. It helps reduce complications associated with inaccurate TBSA representation, such as overcorrection of fluids and the risk of edema due to excess fluid accumulation [16]. Other methods, such as the “rule of nines” and the “rule of palms,” rely on the Lund-Browder Chart [16, 17].

Evaluation of burn scars

POSAS is a subjective determination of scar severity. The patient component consists of six parameters: color, stiffness, scar-related pain, thickness, itchiness, and irregularity. Each parameter uses a 10-point scoring system, with 1 representing normal skin and 60 representing the worst scar imaginable. The VSS rates scars on four parameters: vascularity, pliability, height, and pigmentation. The scar is scored from 0 (normal skin) to 13 (worst scar imaginable) [17]. Other modalities, such as laser Doppler imaging, ultrasound, and thermography, can also be used in scar assessment. Although the gold standard for burn depth assessment is wound biopsy, it is employed with caution, as it may impede the natural healing process of the wound [18].

Non-surgical management

Topical and Intralesional Therapies

Silicone gel and sheets: The use of silicone gel and sheets is a well-established practice in the management of hypertrophic scars and keloids, particularly following burn injuries. Their primary mechanism involves hydrating the stratum corneum through occlusion, reducing transepidermal water loss, and maintaining a moist environment. This hydration influences cytokine-mediated signaling, leading to decreased fibrotic activity. Reduced keratinocyte stimulation lowers fibroblast activity, thereby decreasing collagen deposition and scar hypertrophy. Additionally, the protective barrier minimizes mechanical stress, contributing to improved scar pliability and reduced erythema. Overall, silicone gel promotes balanced hydration and occlusion, optimizing scar outcomes [19]. Clinical research has shown that silicone gel and sheets play a crucial role in enhancing scar healing. A systematic review and meta-analysis revealed that silicone gel notably decreased pigmentation and scar height while improving pliability when compared to placebo or no treatment at all, as evaluated by the VSS. These findings indicate that silicone-based treatments effectively improve both the cosmetic appearance and functional quality of scars [20].

Silicone sheets are worn 12-24 hours daily for at least 2-3 months. They are ideal for flat surfaces due to ease of use and minimal side effects. Silicone gels, applied twice daily, are better suited for irregular areas such as the face and joints, effectively reducing scar volume, inflammation, and improving elasticity over 90 days [21]. However, some individuals in warmer climates experience skin maceration, foul odor, and irritation when using gels [22, 23]. Despite these limitations, silicone products remain a cornerstone in the non-invasive treatment of hypertrophic scars and keloids, valued for their effectiveness and safety. Further high-quality research is necessary to refine treatment guidelines and assess the advantages of combining silicone with other therapeutic approaches [20].

Steroid creams and intralesional steroid injections

Topical steroid creams and intralesional corticosteroid injections are essential in treating hypertrophic scars and keloids, helping to reduce scar thickness and alleviate itching and pain. These scars result from a disorganized extracellular matrix (ECM) deposition caused by an excessive fibroproliferative collagen response. Triamcinolone acetonide (TAC) injections effectively improve scar height, vascularity, and pliability. However, they may cause side effects such as skin atrophy and telangiectasia, particularly at higher doses [24]. A recent systematic review and meta-analysis concluded that the combination of corticosteroids and fluorouracil (5-FU) is more effective in treating and preventing hypertrophic scars and keloids, with fewer side effects [25]. Innovative delivery techniques, such as electronically controlled jet injectors, have demonstrated potential in minimizing injection-related pain and increasing patient comfort, offering a valuable alternative for individuals with needle phobia or significant discomfort from traditional injections [26]. Topical corticosteroid creams help reduce edema and itching due to their anti-inflammatory properties. However, they are generally less effective in reducing scar thickness compared to intralesional injections. To improve delivery and effectiveness, corticosteroid-infused tapes and plasters have been proposed as alternative application methods [27].

Botulinum injections

Botulinum toxin type A (BTX-A) has gained recognition as a potential treatment for hypertrophic scars, keloids, and post-burn contractures, serving as an alternative or complement to conventional therapies. Research indicates that BTX-A is more effective than a placebo and may even be superior to corticosteroid injections in specific outcomes, including scar thickness reduction and improvements in VSS scores [28]. Furthermore, the combination of BTX-A and TAC has demonstrated greater effectiveness than TAC alone, indicating a synergistic effect that enhances treatment outcomes. BTX-A should be considered within the broader framework of multimodal scar management, with treatment selection tailored to individual scar characteristics, patient preferences, and prior treatment responses [29].

Emollients and moisturizers

Emollients and moisturizers contribute to scar management by hydrating the skin and enhancing barrier function, which is particularly beneficial for hypertrophic scars. Research indicates that maintaining proper hydration helps reduce transepidermal water loss, potentially lowering fibroblast activity and minimizing hypertrophic scar development. The most commonly used emollients contain aloe vera or petroleum jelly. While silicone gels and sheets remain the first-line treatments for hypertrophic scars due to their superior occlusive properties, emollients are considered more patient-friendly and cost-effective. Some studies suggest that certain moisturizers provide comparable hydration and occlusion, making them a potential alternative in some cases [20, 30]. Table 2 summarizes topical and intralesional therapies.

**Table 2:** Summary of topical and intralesional therapies. Table created by the authors using references [19-30].

Treatment modality	Indications	Side effects
Silicone gels and sheets	Immature wounds to prevent hypertrophic and keloid scar formation (wounds requiring ≥21 days to heal); reduce itching and hydrate wounds	Skin rash, maceration due to excessive sweating, foul odor, pain, and formation of small hematomas
Moisturizers and emollients	Hydrate wounds, provide barrier protection, and soften the skin	Skin rash, itching, and hives
Topical and intralesional steroid injections	Treatment of hypertrophic and keloid scars	Skin atrophy, telangiectasia, and hypopigmentation
Botulinum injections	Hypertrophic scars, keloids, and post-burn contractures	Redness, edema, tenderness, anaphylactic reactions, and temporary muscle weakness at the treated site

Adjunct therapies

Physical Therapy

Pressure therapy through the use of garments is a common approach for managing hypertrophic burn scars, though its effectiveness remains controversial. A review analyzing 15 studies with 1,179 participants highlighted the uncertainty regarding the effectiveness of pressure-garment therapy (PGT) in preventing hypertrophic scarring after burns. No significant differences were observed in scar appearance, pain, or pruritus when comparing PGT to alternative treatments or no treatment at all [31]. In contrast, a meta-analysis reported that pressure therapy at 15-25 mmHg significantly improved scar characteristics, including thickness, brightness, redness, pigmentation, and hardness, compared to non-pressure or low-pressure treatments. However, the study emphasized the need for further well-designed research to validate these findings and assess potential side effects [32]. When evaluating the combination of pressure therapy and silicone therapy versus either treatment alone, the evidence does not indicate significant differences in overall efficacy. Nonetheless, some studies suggest that combining both therapies may provide advantages in improving scar height and pliability [23].

Laser Therapy

Fractional carbon dioxide (CO₂) laser therapy is an effective scar treatment due to its ability to remodel collagen and enhance dermal elasticity. It involves fractional photothermolysis, which creates microscopic treatment zones that promote faster healing and collagen remodeling. The laser also stimulates neocollagenesis, thickening and tightening the dermal layer, while increasing matrix metalloproteinase 9 (MMP-9) activity to break down disorganized collagen and improve scar pliability [33]. Fractional CO₂ laser therapy significantly reduced scar thickness as measured by ultrasonography. Additionally, improvements were observed in pigmentation, vascularity, pliability, scar height, and relief [34]. Laser therapy provides significant improvements in scar appearance, pain, and pruritus, while maintaining a favorable safety profile and without depending on patient adherence. Combining a fractional CO₂ laser with a pulsed dye laser (PDL) has been shown to enhance outcomes in immature scars. Fractional CO₂ laser combined with BTX-A or topical growth factors has demonstrated superior efficacy in reducing scar thickness and improving VSS scores without increasing adverse effects [35]. Studies also suggest that initiating laser therapy after 12 months post-injury, and using specific lasers such as PDL, can lead to greater improvements in vascularity and scar height reduction [36].

Platelet-Rich Plasma (PRP)

A randomized controlled trial found that PRP combined with fractional CO₂ laser therapy resulted in significant improvements in clinical outcomes, as assessed by VSS scores and other biometric measures [37]. A systematic review also reported that PRP use led to superior scar assessment scores and reduced healing time among burn patients compared to the control group. The incidence of infection and other complications was similar in both groups [38].

Microneedling

Microneedling, also known as percutaneous collagen induction therapy, has demonstrated potential in treating post-burn hypertrophic scars. A study found that combining microneedling with triamcinolone administration significantly improved VSS scores, with superior outcomes particularly in patients with acute scars (less than one year post-burn) [39]. An experimental study in mice demonstrated that a combination of microneedling and PRP resulted in increased collagen content and enhanced scar maturation, potentially accelerating the scar healing process [10].

Cold Therapy

Cold therapy, such as ice pack placement, can be used immediately after superficial burns to reduce edema, pain, and pruritus. However, the beneficial effects last for approximately 30 minutes [40, 41]. A study comparing cooling burn wounds with cold water for 20 minutes versus more than 20 minutes found no significant difference between the two. The optimal duration and temperature for cooling burn wounds currently remain debatable and warrant further research [42].

Surgical management

Surgical management of burn injuries is a complex therapeutic task involving a wide variety of procedures that often require multiple interventions over time to achieve both functional and aesthetic outcomes. Surgical interventions include direct wound closure, tissue excision, skin grafting, and flap procedures. Surgical reconstruction of burn injuries is generally divided into primary reconstruction and secondary reconstruction. Primary reconstruction takes place immediately after the burn injury, while secondary reconstruction occurs once scar formation has developed. A hemodynamically stable patient may benefit from early interventions such as free flaps, as a significant increase in coagulability after 48 hours of burn injury is a concern. However, patients with extensive burn injuries require immediate fluid resuscitation before undergoing surgical procedures due to burn shock, which is characterized by distributive shock and fluid shifts in the early stage of trauma [43].

Surgical procedures are not typically performed in the acute setting unless essential function is compromised. Definitive reconstruction is usually carried out one year after the injury. Indications for early surgical intervention include airway compromise, eyelid release to protect an exposed cornea, correction of distracted or entrapped neurovascular bundles, severe fourth-degree contractures, and severe microstomia [44]. Such complications are frequently managed with wound excision techniques. Early wound excision, debridement, and skin grafting remain the standard of care for burn wounds to prevent scarring. The main objective of early debridement is to remove necrotic tissue, reduce the risk of infection, and optimize the wound bed for healing. Direct wound closure refers to the use of staples or sutures to approximate wound borders. This is indicated for small to moderately sized wounds that are suitable for excision of necrotic and infected tissue. Its main advantage is replacing burned skin with a more aesthetic closure scar. However, direct closure is not recommended for wounds that cross or are near joint lines. Moreover, it has been associated with higher rates of infection, dehiscence, and abnormal scarring [43-45].

**Figure 2:**
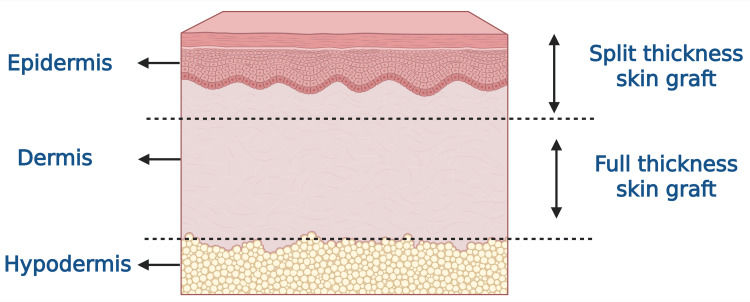
Types of skin grafts. Image Credit: Figure created by Humza Siddiqui using BioRender.com.

Skin grafting is the most frequently employed surgical method in burn management, according to the Japanese Society for Burn Injuries. Allogenic skin grafts are considered the gold standard for extensive burn treatment worldwide [45]. Tangential excision followed by skin grafting is a fundamental technique in burn surgery, as it maintains dermal vascularity and integrity, thereby reducing the risk of excessive scarring [46]. The choice of debridement technique also influences scar quality. Hydrosurgical debridement has been shown to preserve more dermal tissue than traditional techniques, leading to improved scar pliability and overall quality 12 months post-surgery [47]. One study reported a significant difference between graft techniques, with improved scar height and flexibility at six months when using the overlapping graft edges technique [48]. The decision to use grafts depends on the depth and location of the wound. The standard technique includes autologous split-thickness skin grafts [49]. Figure 2 illustrates the types of skin grafts.

These autografts require a sterile operative site before closure. They can be meshed to allow expansion and drainage. Sheet grafts are commonly used for aesthetic reasons [2]. Skin grafting is indicated for deep partial-thickness (second-degree) and full-thickness (third-degree) burns, traumatic injuries, and extensive or non-healing wounds that cannot close through primary or secondary intention. Conditions such as diabetes mellitus, chronic vascular disease, and immunocompromised states are often considered contraindications for dermal substitutes [50]. A study comparing early grafting (within 5 days of injury) to delayed grafting (after 3 weeks) found slightly higher blood loss and transfusion requirements in the early group, but significantly shorter hospital stays [51]. Newer autologous skin grafts tend to be more expensive and require longer hospitalization compared to traditional mesh grafts [52]. Acellular fish skin xenografts are gaining popularity for burn wound management, as they improve both functional and aesthetic outcomes [53]. Both bioengineered acellular fish skin matrix (BAM) and natural fish skin (NFS) have shown favorable outcomes in reducing pain and accelerating healing, although NFS offered superior cost benefits [54]. Tilapia skin biomaterial has also demonstrated potential as an effective burn wound dressing [55].

The fundamental difference between grafts and flaps lies in their vascular supply. Flaps maintain their own blood supply, allowing modern surgical techniques to reconstruct large and complex wounds. Their main limitation is the limited availability of viable donor tissue in burn patients. Flaps are classified based on their blood supply, the proximity of donor and recipient tissue, and tissue composition [56]. Commonly used flaps in burn reconstructive surgery include transposition flaps, pedicled gastrocnemius flaps, free anterolateral thigh flaps, and free latissimus dorsi flaps [57]. Transposition flaps, a type of local flap, mainly include Z-plasty and W-plasty. Local flaps are less likely to contract because they naturally expand after surgery, unlike skin grafts. Both plasty techniques consist of raising two or more opposing flaps and transposing them along a shared axis, creating a final Z-like figure on the wound site. Z-plasty is primarily used to lengthen and reorient a scar for either functional or aesthetic purposes. Its key benefit is faster scar healing compared to linear scars. It is often employed to release scar tissue contracture, stenosis, and motion-limiting scarring. Common contraindications for Z-plasty include keloid or hypertrophic scarring tendencies as well as defective wound healing. Possible complications include hematoma, venous congestion, necrosis, worsened scarring, or dehiscence. W-plasty is not as frequently used, as Z-plasty is generally less complicated and can be applied in a wider variety of skin locations. The main advantage of W-plasty is its poor reflection of light, making scars less visible on flat surfaces such as the cheek. However, W-plasty is not indicated when wounds are located on major joints such as the elbow [58]. Perforator-based interposition flaps are superior to full-thickness skin grafts in terms of surface area, range of motion, and elasticity, making them a better therapeutic option for secondary burn reconstruction, particularly in joints and the anterior neck [59-61]. When harvested, free flaps are fully separated from the donor site and transplanted to a distant body region, also known as an “island flap.” The latissimus dorsi flap, anterolateral thigh flap, and radial forearm flap are among the free flaps commonly used in burn reconstruction. In the context of primary reconstruction, free flaps can accomplish limb salvage as well as one-stage repair of complex burn defects with optimal results and low morbidity. Fascio-cutaneous free flaps are associated with fewer complications and better outcomes compared to muscle flaps in acute burn injury reconstruction. Free flaps are particularly useful for reconstruction of the hand and foot, where thin soft tissue easily exposes nerves, tendons, and bone. Bony prominences such as the knee, tibia, and elbow also benefit from free flap reconstruction because of their thin skin coverage [62].

More than 50% of burn patients in developing countries experience post-surgical complications [63]. Severe cases in these regions report mortality rates as high as 41% [64]. According to the American Burn Association, in the United States, the mortality rate is 2.6% among patients with deep burns requiring surgical management without prolonged ventilation, and 17.8% among those requiring surgical management with prolonged mechanical ventilation. In terms of patient satisfaction, burn reconstruction surgery provides significant improvements in both function and quality of life [65].

Emerging therapies

Stem Cell Therapy (SCT)

SCT is an emerging technique aimed at treating burn scars by leveraging the ability of progenitor cells to differentiate into various cell types necessary for skin repair. SCT is particularly effective for second-degree burns, with various types of transplants used, including xenogeneic, allogeneic, and autologous [66]. The most common cell types include mesenchymal stem cells (MSCs), commonly derived from bone marrow and human umbilical cord, hair follicle stem cells (HFSCs), and adipose-derived stem cells (ASCs) [67]. According to a meta-analysis of 22 studies involving 595 animals, HFSCs were the most effective type of stem cells for treating burn wounds. However, this analysis focused primarily on animal models with minimal human implications. MSCs were the most frequently used in human studies and clinical settings [68].

A clinical trial evaluated the efficacy and safety of bone marrow-derived mesenchymal stem cells (BM-MSCs) in treating burn scars. Ten patients (≥18 years) with deep second-degree burns (≤20% TBSA) participated. These patients received two BM-MSC doses (2.5 × 10³ BM-MSC/cm² and 5 × 10³ BM-MSC/cm²), applied either topically or subcutaneously under a transparent film dressing within seven days of injury, and were monitored for six months. Healing outcomes were assessed based on wound closure rate (cm²/day), POSAS for scar quality and healing, and cytokine analysis for immune response monitoring. The study found that wound closure was achieved in all patients, with no adverse reactions or immune rejection observed, as cytokine levels remained stable pre- and post-treatment. Minimal to no fibrosis was observed with the pinch test. Re-pigmentation and hair follicle restoration were noted in some patients, and POSAS scores improved over time [69].

Nanomedicine 

Nanomedicine is an emerging approach for managing burn scars through the use of organic and inorganic nanoparticles. Their small size enables penetration into different tissues, providing a distinctive advantage over traditional burn scar treatments. The main types of nanomedicine studied for burn scars include polymeric nanoparticles, metal nanoparticles, nanogels, and nanoemulsions. Polymeric nanoparticles, composed of biodegradable polymers such as polylactic acid (PLA) or polyglycolic acid (PGA), are used to deliver drugs, growth factors, and other therapeutic agents directly to target tissues. Silver and gold nanoparticles are the most widely studied metallic forms, valued for their antimicrobial, anti-inflammatory, and antioxidant properties. Nanogels are hydrophilic polymer networks capable of sequestering therapeutic agents, thereby amplifying scar healing. Nanoemulsions, which are lipid-based systems, have demonstrated both antibiotic and anti-inflammatory effects [70]. The use of synthesized silver nanoparticle (AgNP) ointment accelerated wound closure and reduced inflammation by suppressing pro-inflammatory cytokines such as interleukin (IL-6, IL-1β) and tumor necrosis factor (TNF-α), while increasing anti-inflammatory markers such as IL-10. Furthermore, the ointment promoted collagen deposition, reduced mast cell migration, and enhanced re-epithelialization, resulting in accelerated healing compared to untreated wounds [71]. Nanogels loaded with metal oxide particles have demonstrated strong antibacterial activity against both Gram-positive and Gram-negative bacteria, thereby accelerating wound healing, tissue regeneration, and controlling inflammation in in vivo models. Histological analysis confirmed the promotion of granulation tissue formation, neovascularization, collagen deposition, and re-epithelialization [70].

3D Skin Bioprinting

3D skin bioprinting is an advanced tissue-engineering technique that uses 3D printing technology to deposit bioinks (hydrogel-based scaffolds) typically composed of living cells, human plasma, fibroblasts, keratinocytes, biomaterials, and growth factors in a precise layer-by-layer manner to create a structure that replicates the skin’s architecture by mimicking the natural ECM and supporting cell viability and differentiation. This technology can be applied in two main ways: in situ bioprinting, where the bioprinter directly deposits pre-cultured cells and scaffolds onto the burn site to enable real-time tissue regeneration, and in vitro bioprinting, where skin constructs are printed and later transplanted onto the patient [72].

One study demonstrated the successful regeneration of 3D-printed skin with properties identical to human skin. The 3D skin consisted of a dermal layer containing a fibrin matrix with fibroblasts and an epidermal layer containing keratinocytes. In vitro analysis of the skin showed complete epidermal differentiation and the presence of essential skin markers, such as K10 in suprabasal cells and vimentin in fibroblasts, confirming proper tissue formation. The 3D-printed skin was transplanted into immunodeficient mice, and in vivo analysis further validated its functionality, as the skin developed all native human skin layers, including rete ridges and a well-developed dermo-epidermal junction marked by collagen VII. Neoangiogenesis was also observed, ensuring long-term tissue viability [73]. Another preclinical study, evaluating the therapeutic efficacy of 3D-printed artificial skin using a mouse chimney model, found that 3D-printed skin substitutes containing porcine-derived decellularized ECM (dECM), human keratinocytes, and fibroblasts demonstrated faster skin regeneration, increased collagen deposition, and reduced inflammatory response compared to the control group. These findings were validated through histological and immunohistochemistry analysis [74]. Although 3D skin bioprinting holds significant potential, it remains an emerging technology currently undergoing clinical evaluation. The Centre for Burn Research at Hamilton Health Sciences has recently initiated a clinical trial to assess the safety and efficacy of 3D bioprinted skin for extensive burns. This trial aims to address critical challenges such as the scalability of bioprinted skin and its integration with host tissue [75].

## Conclusions

Burn cases pose a significant burden on healthcare systems globally. Acute burn injuries can result in severe fluid shifts, shock, and sepsis. In the long term, they may lead to the formation of hypertrophic scars and contractures, which impair function and aesthetic appearance. Conventional strategies include the application of emollients, corticosteroids, and silicone gels for managing superficial and partial-thickness burns. Surgical procedures such as flaps and grafts are employed for deep partial-thickness and full-thickness burns. Newly emerging techniques have also shown promising results with superior outcomes. It is imperative to establish definitive treatment guidelines and further explore emerging therapies to provide maximum benefit and enhance patients’ quality of life.
